# Comparison of clinically approved molecules on SARS-CoV-2 drug target proteins: a molecular docking study

**DOI:** 10.3906/kim-2008-35

**Published:** 2021-02-17

**Authors:** Hasan ÇUBUK, Mehmet ÖZBİL

**Affiliations:** 1 Department of Molecular Biology and Genetics, Faculty of Arts and Sciences, İstanbul Arel University, İstanbul Turkey; 2 Institute of Biotechnology, Gebze Technical University, Kocaeli Turkey

**Keywords:** SARS-CoV-2, COVID-19, furin protease, remdesivir, molecular docking

## Abstract

The new type of coronavirus, SARS-CoV-2 has affected more than 22.6 million people worldwide. Since the first day the virus was spotted in Wuhan, China, numerous drug design studies have been conducted all over the globe. Most of these studies target the receptor-binding domain of spike protein of SARS-CoV-2, which is known to bind to the human ACE2 receptor and SARS-CoV-2 main protease, vital for the virus’ replication. However, there might be a third target, human furin protease, which cleaves the virus’ S1-S2 domains playing an active role in its entry into the host cell. In this study, we docked five clinically used drug molecules, favipiravir, hydroxychloroquine, remdesivir, lopinavir, and ritonavir onto three target proteins, the receptor-binding domain of SARS-CoV-2 spike protein, SARS-CoV-2 main protease, and human furin protease. Results of molecular docking simulations revealed that human furin protease might be targeted by COVID-19. Remdesivir, a nucleic acid derivative, strongly bound to the active site of this protease, suggesting that this molecule can be used as a template for designing novel furin protease inhibitors to fight against the disease. Protein-drug interactions revealed in this study at the molecular level, can pave the way for better drug design for each specific target.

## 1. Introduction

Coronaviruses are a type of single-stranded RNA virus that infects mammals and birds. In humans, they cause respiratory diseases ranging from the common cold to severe/fatal illnesses [1]. Three types of human-infecting coronaviruses have been associated with deadly phenomena since the early period of the 2000: severe acute respiratory syndrome coronavirus (SARS-CoV), Middle-East respiratory syndrome coronavirus (MERS-CoV), and severe acute respiratory syndrome coronavirus 2 (SARS-CoV2). In November 2002, SARS-CoV affected 8098 people and caused 774 deaths in China in the period up until June 2003. In June 2012, MERS-CoV appeared in the Middle East, resulting in over 2000 cases, and by 2017 about 600 deaths were reported [2,3].

SARS-CoV-2, discovered in China, has affected over 22,639,650 people and killed 792,197 in more than 215 countries as of August 20, 2020.1World Health Organization (2020) onward (continuously updated). WHO Coronavirus Disease (COVID-19) Dashboard [online]. https://covid19.who.int/?gclid=CjwKCAjw8df2BRA3EiwAvfZWaP34yJr8HdK4mBed5dKa2T6flZjBA5sFDNCata6LM6-eXa1CmMjHwhoCUZQQAvD_BwE [accessed 01 June 2020]. The World Health Organization (WHO) announced “COVID-19” as the name of a new disease caused by SARS-CoV-2.2World Health Organization (2020) onward (continuously updated). WHO Coronavirus Disease (COVID-19) Dashboard [online]. https://www.who.int/emergencies/diseases/novel-coronavirus-2019/technical-guidance/naming-the-coronavirus-disease-(covid-2019)-and-the-virus-that-causes-it [accessed 01 June 2020]. The ongoing COVID-19 threat rapidly spread all over the globe and is still transfecting humans. Thus, many efforts are ongoing in the investigation of suitable preventive and control strategies, as neither vaccines nor direct-acting antiviral drugs are available for the treatment of human SARS-CoV-2.

Most of the therapeutic options for COVID-19 were based on antiviral agents, which are used for treating previous Zika, Ebola, and Nipah viruses, SARS-, and MERS-CoVs [4]. This is due to the fact that the time required for drug discovery programs to develop, evaluate, and obtain appropriate new therapeutic agents might take more than 10 years. Thus, researchers are focused on therapeutics, which have proven efficacy against viruses similar to COVID-19 instead of a new potent anti-COVID-19 agent. These available therapeutic agents against SARS-CoV-2 could be either virus-based, involving small molecules targeting viral S protein, viral protease inhibitors, and RBD–ACE2 blockers, or host cell-based including host cell protease inhibitors and host cell endocytosis inhibitors [4].

Spike protein directly mediates viral entry with the S1 domain, which is responsible for host cell surface binding through ACE2 receptors and S2 domain which is responsible for membrane fusion. The viral binding to host cell surface follows S1/S2 cleavage by host proteases such as TMPRSS2, and cathepsins B and L. Previous studies have already demonstrated that furin, a proprotein convertase, can mediate S1/S2 cleavage unlike other coronaviruses and contribute to membrane fusion efficiency which explains the current strong infectious capacity of SARS-CoV-2 [2,3]. Thus, SARS-CoV-2^RBD^/ACE2 and Furin could be potential targets for COVID-19 to prevent viral entry. Furthermore, SARS-CoV-2 main protease known as M^pro^, which is essential in processing viral polyproteins and viral replication, could be a nontoxic target for managing COVID-19 as humans do not have proteases with similar cleavage specificity [5].

In vitro studies by Liu et al. had already demonstrated that two drugs, chloroquine (CQ) and hydroxychloroquine (HCQ) efficiently inhibited SARS-CoV-2 infection in vitro and these findings were also supported by preliminary clinical studies [6–9]. Several other drugs such as remdesivir and favipiravir are currently undergoing clinical studies to test their efficacy and safety in the treatment of COVID-19 in China and other European countries such as Turkey, and some promising results have been achieved so far 3Republic of Turkey Ministery of Health. (2020) onward (continuously updated). Favipiravir 200 mg Tablet - COVID-19 (SARS-CoV2 Enfeksiyonu) Tedavisinde Kullanılacak İlaçlara İlişkin Bilgilendirme [online]. Website: https://khgmstokyonetimidb.saglik.gov.tr/TR,64669/favipiravir-200-mg-tablet---covid-19-sars-cov2-enfeksiyonu-tedavisinde-kullanilacak-ilaclara-iliskin-bilgilendirme.html. [accessed 01 June 2020].[10 – 13]. Furthermore, lopinavir and ritonavir are widely used as HIV protease inhibitors, and previous in vitro and in vivo studies have also shown their potential activity against other coronaviruses; SARS-, and MERS-CoVs [14–16].

In the present study, we investigated the binding of five active molecules, currently applied as the first line of treatment, favipiravir, HCQ, remdesivir, lopinavir, and ritonavir onto three different possible target proteins, the receptor-binding domain of SARS-CoV-2 spike protein (SARS-CoV-2^RBD^), SARS-CoV-2 main protease (SARS-CoV-2 M^pro^), and human furin (hFUR) protease by molecular docking simulations. We aimed to shed light on the functional group selection for future drug design studies by reporting protein-drug interactions at the molecular level. 

## 2. Materials and methods

### 2.1. Preparation of the protein and target molecules

The crystal structure of the human proprotein convertase furin (PDB: 5JXG,1.8 Å) [17], COVID-19 main protease in complex with an inhibitor N3 (PDB: 6LU7, 2.2 Å) [18], without inhibitor (APO form) (PDB ID: 6M03, 2.0 Å) by Zhang et al, which has not yet been published, and coronavirus spike receptor-binding domain complexed with its receptor ACE2 (PDB: 6LZG, 2.5 Å)[19] were obtained from the Research Collaboratory for Structural Bioinformatics Protein Data Bank (RCSB PDB). Small molecules were removed from crystal structures by using BIOVIA Discovery Studio software (BIOVIA, San Diego, CA, USA) [20].4Dassault Systèmes BIOVIA, Discovery Studio Modeling Environment, Release 2017, San Diego: Dassault Systèmes, 2016. Polar hydrogens and Kollman charges were added to the protein and a pdbqt format file was generated using AutoDockTools 1.5.6 software (Scripps Research, San Diego, CA, USA) [20].

The canonical simplified molecular-input line-entry system (SMILES) of lopinavir, remdesivir, HCQ, favipiravir, and ritonavir was obtained from the PubChem database. Their structures were built, and structural minimization was carried out on UCSF Chimera software (RBVIu1d35, CA, USA) to obtain a stable conformer [21]. Structural minimizations were directly handled through default parameters on UCSF Chimera u1d38, CA, USA) software (except for applying Gasteiger charges) to minimize molecules. Afterward, the structures were converted into pdbqt format using AutoDockTools 1.5.6 software (San Diego, CA, USA), for use for docking calculations with AutoDock Vina (San Diego, CA, USA).

### 2.2. Docking 

Molecular docking simulations are a widely used computational method for the initial guess of ligand binding to receptor protein [22]. AutodockVina 1.1.2 software (San Diego, CA, USA) [23] was used for docking calculations and the exhaustiveness parameter was selected as 8, and 10 models were generated for each ligand. Windows 7 Ultimate operating system (64-bit), installed on a home-built desktop computer, equipped with Intel Core i3-3110M 2.40 GHz processor and 8GB memory, was utilized for all computational work. Results were analyzed using BIOVA Discovery Studio software (San Diego, CA, USA) and VMD-Visual Molecular Dynamics software (The University of Illinois, Champaign, IL, United States)  [24].

## 3. Results

Due to the recent COVID-19 pandemic, many efforts have been directed to the investigation of suitable preventive and control strategies for the treatment of human SARS-CoV-2 infection. Almost all these attempts are with the previously used antiviral agents, such as lopinavir, ritonavir, favipiravir, HCQ, and remdesivir. To unravel mechanism and inhibition capability of these five clinically used drugs against SARS-CoV-2 infections, we have run molecular docking simulations using three therapeutic targets; SARS-CoV-2^RBD^-ACE2 interface, SARS-CoV-2 M^pro^, and hFUR. 

**Figure 1:**
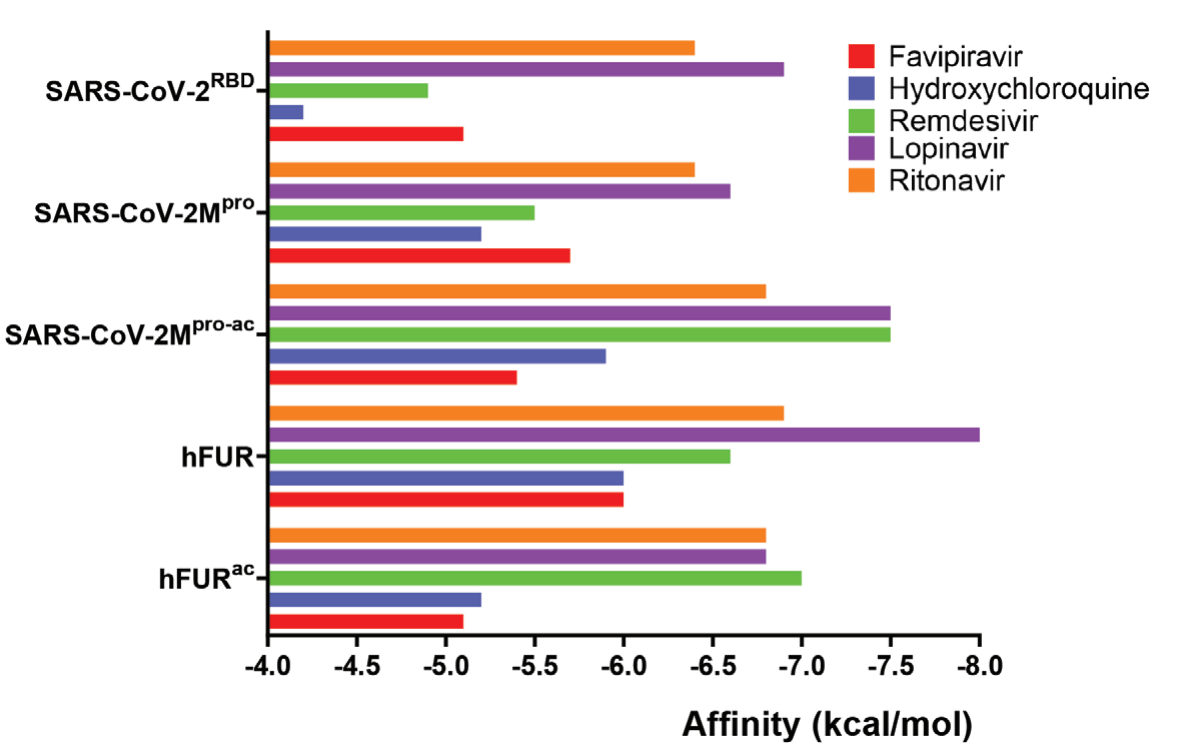
Graphical representation of binding affinities for five molecules on three proteins including the active site of proteases, SARS-CoV-2-M^pro-ac^ and hFUR^ac^.

**Figure 2:**
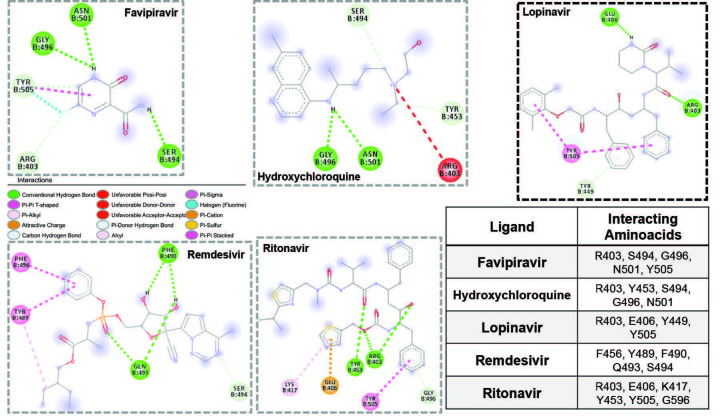
Molecular interactions for five molecules at the SARS-CoV-2^RBD^ site. Drug molecule with the highest binding affinity was highlighted in bold frame. Legend for interactions was provided in the middle and interacting amino acid residues were provided in the table.

First, we designed docking analyses with a grid box covering only the SARS-CoV-2^RBD^-ACE2 interface. The binding of five molecules onto the SARS-CoV-2^RBD^ yielded binding affinities ranging from –4.2 kcal/mol to –6.9 kcal/mol (Table and Figure 1). Among these active molecules, lopinavir and ritonavir yielded higher binding affinities towards this target, –6.9 kcal/mol and –6.4 kcal/mol, respectively. SARS-CoV-2^RBD^ residues Arg 403 created hydrogen bonds with both ligands, Tyr 453 made H-bond with ritonavir, Glu 406 interacted with lopinavir through hydrogen bonds and with ritonavir through electrostatic interactions, and *Tyr 505* was involved in π-π interactions in both cases. Favipiravir, HCQ, and remdesivir were not potential binders of the SARS-CoV-2^RBD^-ACE2 interface despite creating hydrogen bonds with RBD residues Arg 403, Tyr 453, Phe 490, Gln 493, Ser 494, Gly 496, and Asn 501. Relatively small sizes of favipiravir and HCQ must be the reason for small binding affinities obtained in the study (Figure 2). Thus, these drugs are less likely to bind and inhibit the SARS-CoV-2^RBD^-ACE2 interface during SARS-CoV-2 infection.

**Table:** TBinding affinities in kcal/mol of five molecules on three different target proteins.

Binding affinities (kcal/mol)	Favipiravir	Hydroxychloroquine	Remdesivir	Lopinavir	Ritonavir
SARS-CoV-2^RBD^	–5.1	–4.2	–4.9	–6.9	–6.4
SARS-CoV-2 M^pro-ac^	–5.4	–5.9	–7.5	–7.5	–6.8
SARS-CoV-2 M^pro^	–5.7	–5.2	–5.5	–6.6	–6.4
hFUR^ac^	–5.1	–5.2	–7.0	–6.8	–6.8
hFUR	–6.0	–6.0	–6.6	–8.0	–6.9

**Figure 3:**
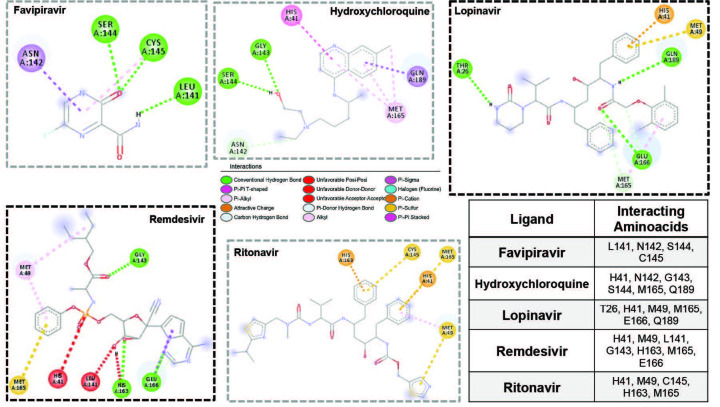
Molecular interactions for five molecules at the SARS-CoV-2M^pro-ac^ site. Drug molecule with the highest binding affinity was highlighted in bold frame. Legend for interactions was provided in the middle and interacting amino acid residues were provided in the table.

Secondly, we set out docking simulations to examine the binding of these drugs on SARS-CoV-2 M^pro^, another very important element in COVID-19 infection. Docking analysis onto SARS-CoV-2 M^pro^ didn’t yield significantly higher binding affinities than those for SARS-CoV-2^RBD^, ranging from –5.2 kcal/mol to –6.6 kcal/mol. Lopinavir was bound with the highest binding affinity, i.e. –6.6 kcal/mol (Table and Figure 1). All the binding sites for all five drug molecules were outside the active site of the protease. Therefore, we repeated simulations with smaller simulation box, targeting only the active site, SARS-CoV-2 M^pro-ac^, comprised of amino acid residues Thr 26, His 41, Met 49, Leu 141, Asn 142, Gly 143, Ser 144, Met 165, Glu 166, and Gln 189. Molecular interactions at this site are provided in Figure 3. This time binding affinities increased for all ligands except for favipiravir. Specifically, for lopinavir and remdesivir binding affinities increased by 0.9 kcal/mol and 2.0 kcal/mol, respectively and both active molecules produced binding affinities of –7.5 kcal/mol. Active site residues on the SARS-CoV-2 M^pro^ (His 41, Met 49, and Met 165) were active interactions, and Thr 26, Glu 166, and Gln 189 interacted through hydrogen bonds with lopinavir. Similarly, active site residues His 41, Met 49, Leu 141, and Met 165 were actively involved in binding and Gly 143, His 163, and Glu 166 were involved in hydrogen bonding with remdesivir, proving that both ligands could have their own inhibitor activity through these active site residues by a direct active site blockage. Also, high binding affinities of lopinavir and remdesivir were probably due to interactions between His 41, Met 49, and Met 165 residues, and aromatic rings present on drug molecules thereby, drugs targeting the active site of SARS-CoV-2 M^pro^ should include aromatic rings to obtain high binding affinity. As a consequence, our data suggest that lopinavir may also target SARS-CoV-2 M^pro^ as it is an antiretroviral protease inhibitor of HIV-1. Hence, this would be an explanation for the clinical activity of lopinavir against SARS-CoV-2. This binding site was also predicted to be a lopinavir binding site in a computational study by Liu et.al. [16] and was also revealed in the X-ray structure (PDB ID: 6LUV) [18] for a peptide derivative inhibitor N3 (Figure SI-1). We also investigated the recently revealed crystal structure of SARS-CoV-2 M^pro^ in its APO form by Zhang et al. (PDB ID: 6M03), which has not yet been published. Molecular docking simulations produced very similar results compared to the inhibitor bound crystal structure. For whole protein docking, lopinavir was still clearly the best ligand, owing to the highest binding energy. The binding energy of ritonavir and favipiravir slightly increased, while that of HCQ, lopinavir, and remdesivir declined. The energy difference of active molecules on both APO and HALO state was in the range of ±0.7 kcal/mol (Figure SI-2). When molecular docking simulations were repeated for SARS-CoV-2 M^pro-ac^, binding affinities for all ligands slightly increased, however, the binding affinity order did not change. Lopinavir and remdesivir were still the best binding ligands to the active site of SARS-CoV-2 M^pro^ (Figure SI-2). These similar results were not surprising upon utilizing SARS-CoV-2 M^pro^ in its APO form, rather than inhibitor bound HALO form, because both crystal structures superimposed very well with an RMSD value of 0.49 Å (the most unmatched region was the C-terminal), which proved very similar target protein structures for both types (Figure SI-3). 

**Figure 4:**
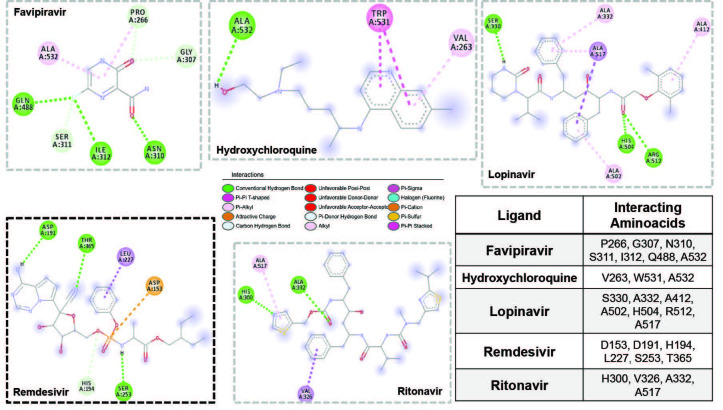
Molecular interactions for five molecules at the hFUR. Drug molecule hitting the active site directly was highlighted in bold frame. Legend for interactions was provided in the middle and interacting amino acid residues were provided in the table.

The last target protein investigated was hFUR. Binding affinities obtained were in the range of –6.0 kcal/mol to –8.0 kcal/mol (Table and Figure 1). Lopinavir provided the highest binding affinity with –8.0 kcal/mol among all active molecules. On the other hand, ritonavir and remdesivir bound to this target with binding affinities of –6.9 kcal/mol and –6.6 kcal/mol, respectively. Molecular interactions between all ligands and hFUR are provided in Figure 4. As it can be seen from the table in Figure 4, remdesivir was the only active molecule to hit the active site directly, even though the grid box covered the whole protein (Figure SI-4). We repeated docking simulations with a smaller grid box covering only the active site of human furin (hFUR^ac^), comprised of amino acid residues Asp 153, Arg193, His 194, Arg 197, Leu 227, Val 231, Ser 253, Asp 258, Asn 295, and Ser 368. Binding affinity for remdesivir increased by 0.4 kcal/mol, while it decreased for favipiravir by 0.9 kcal/mol, HCQ by 0.8 kcal/mol, lopinavir by 1.2 kcal/mol, and ritonavir by 0.1 kcal/mol, when compared to docking on whole protein structure. The only increase in binding affinities was observed for remdesivir upon covering only the active site of the protein. Remdesivir dominantly interacted with hFUR^ac^ by the active site of human furin residues Asp 191, His 194, Ser 253, and Thr 365 through hydrogen bonding. Also, hydrophobic interactions between aromatic rings, the phosphoric backbone of remdesivir, and Asp 153 and Leu 227 residues were probably the main reason for high binding affinity. The highly hydrophilic and aromatic character of this pocket also led to high binding affinity (Figure SI-5). This suggests that hFUR could be a main target for the remdesivir even if it involves the inhibition of replication of the viral genome and viral assembly. We should also mention that when all molecules were docked onto the whole hFUR, four molecules, favipiravir, HCQ, ritonavir, and lopinavir didn’t hit the active site. This suggested that hFUR^ac^ may not be accessible to these molecules, but to remdesivir.

## 4. Discussion

In this study, we investigated the binding of readily prescribed drug molecules favipiravir, HCQ, remdesivir, lopinavir, and ritonavir onto three therapeutic targets which should be targeted for COVID-19 treatment. Among all targets, the receptor-binding domain of SARS-Cov2 spike protein (SARS-CoV-2^RBD^), SARS-CoV-2 main protease (SARS-CoV-2 M^pro^), and human furin (hFUR) protease, binding affinities for all drug molecules were calculated to be higher for active sites of both hFUR and SARS-CoV-2 M^pro^. Moreover, remdesivir directly hit the active site of hFUR, with a binding affinity of -7.0 kcal/mol, while other drugs did not target the active site of hFUR directly. The reasons for this direct targeting are the adenosine triphosphate moiety of remdesivir, making strong hydrophobic and polar interactions in the active site supported by hydrogen bonds. 

Clinically used drug molecules; lopinavir, remdesivir, and ritonavir yielded higher binding affinities for both active sites of hFUR and SARS-CoV-2 M^pro^, than other therapeutic targets such as SARS-CoV-2^RBD^ while HCQ and favipiravir yielded less affinity for these therapeutic targets using molecular docking simulations. In addition, remdesivir was a strong binder of the active site of hFUR. Thus, this should be a major reason for the potential activity of remdesivir against COVID-19 in the preclinical studies. hFUR could be targeted for the treatment of SARS-CoV-2 infections other than usual drug targets such as SARS-CoV-2 M^pro^ and SARS-CoV-2^RBD^. Moreover, remdesivir could be targeting both viral replication machines and host proteases responsible for the SARS-CoV-2 entry into cells. Our results indicated that to design hFUR^ac^ inhibitors, remdesivir, a nucleic acid derivative, should be used as a template. Likewise, to target SARS-CoV-2^RBD^ or SARS-CoV-2 M^pro^, a lopinavir like drug molecule should be designed. These findings will lead to more target-specific drug design studies.

Supplementary Materials
