# Supplementary material for: Comparison of clinically approved molecules on SARS-CoV-2 drug target proteins: a molecular docking study
Source: Turk J Chem. 2021 Feb 17;45(1):35–41. doi: 10.3906/kim-2008-35 (PMC7925319; doi:10.3906/kim-2008-35)
Supplement: Supplementary file 1 — Supplementary Materials [file turkjchem-45-35-sup001.pdf]

Supporting Information Figures:

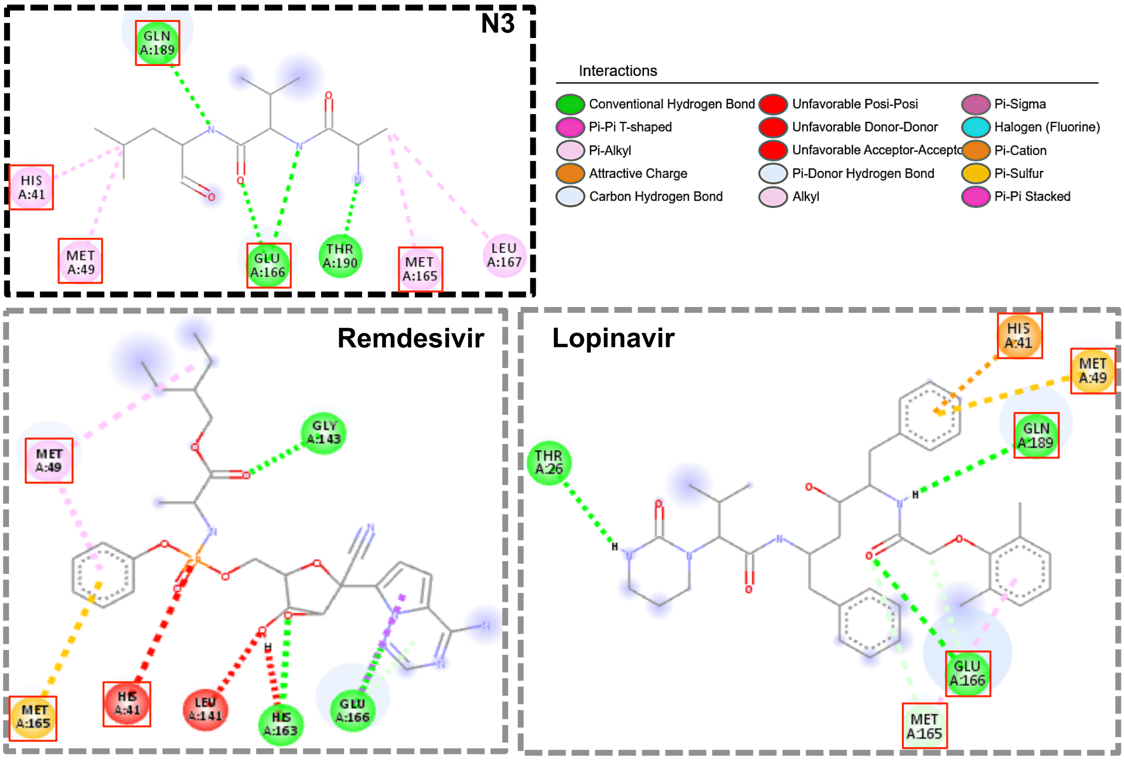

Figure SI-1. Comparison of N3 binding from crystal structure, PDB ID: 6LUV, and remdesivir and lopinavir binding from the current study at SARS-CoV-2 $M^{pro\text{-}ac}$. The same interacting amino acid residues were shown in red rectangular boxes.

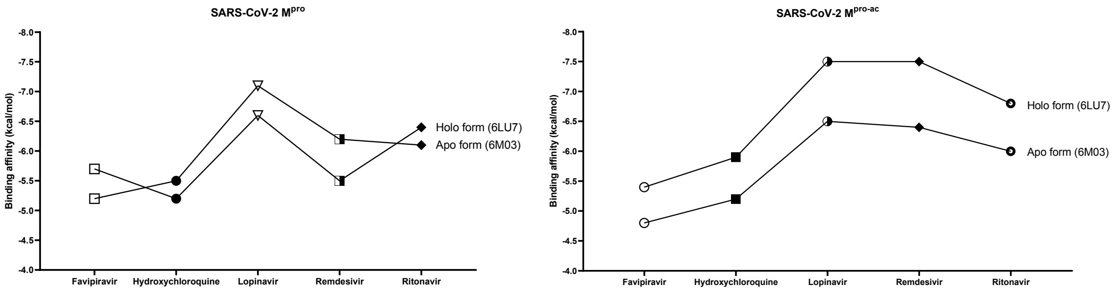

Figure SI-2. Comparison of binding affinities from molecular docking simulations of all ligands for two forms of SARS-CoV-2 $M^{pro/pro\text{-}ac}$ proteins (holo form-PDB ID:6LU7 and apo form-PDB ID:6M03).

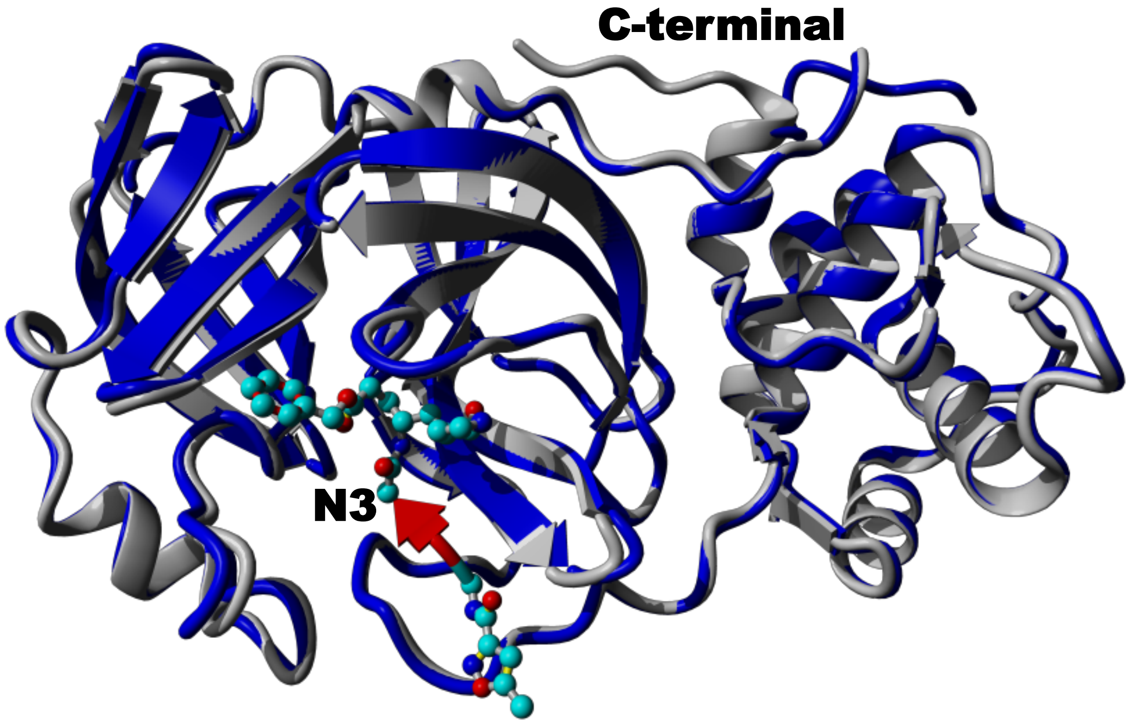

Figure SI-3. Superimposition of crystal structures for two forms of SARS-CoV-2 $M^{pro/pro-ac}$ proteins. Holo form (PDB ID:6LU7) was in blue, inhibitor molecule was represented as ball and sticks and apo form (PDB ID:6M03) was in gray. Mainly different, C-terminal regions were labeled.

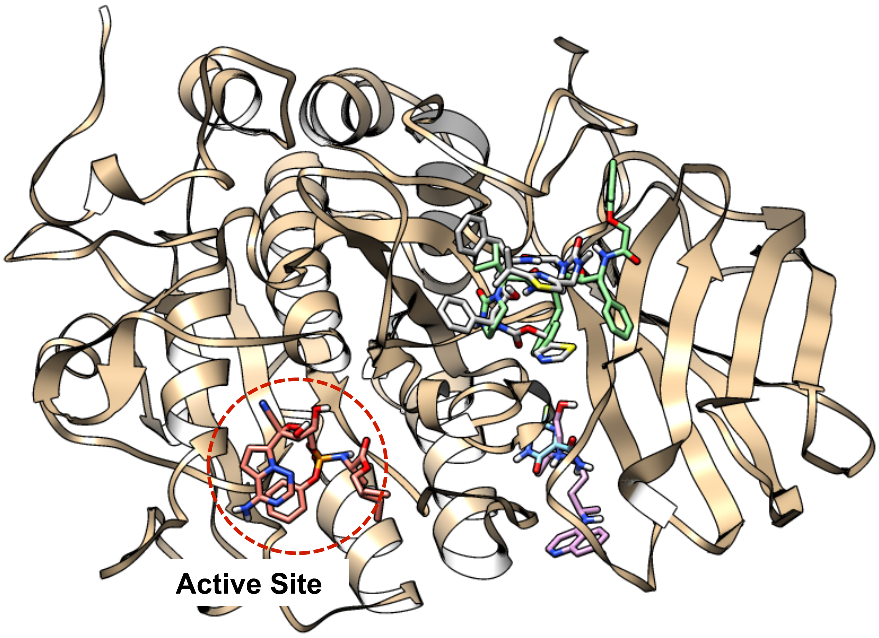

Figure SI-4. Molecular docking simulations results for hFUR. Remdesivir was the only active molecule hitting the active site, although the simulations grid box covered the entire protein.

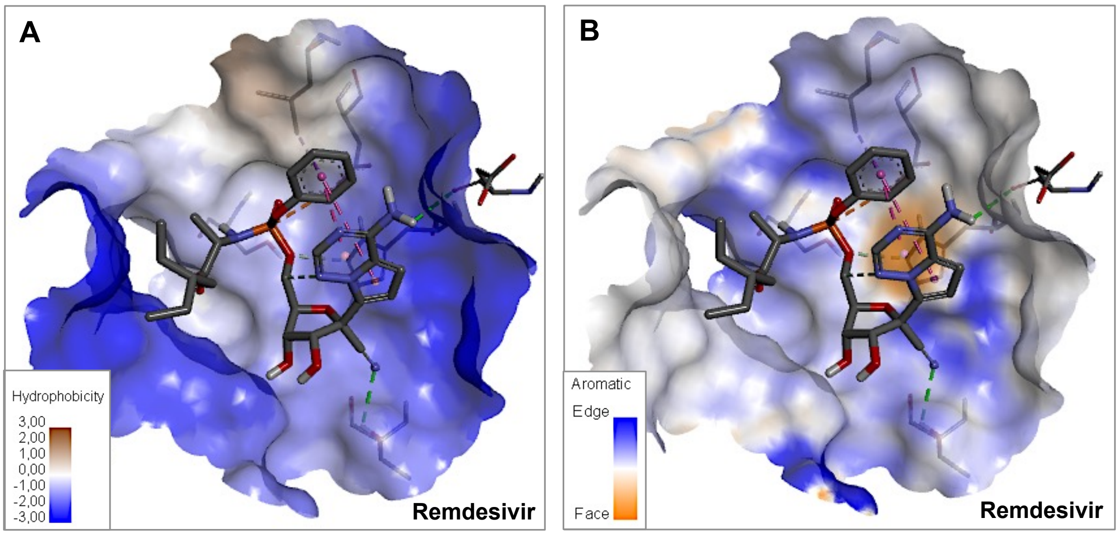

Figure SI-5. A) Hydrophobicity and B) aromaticity of hFUR[ac]. The active site is highly hydrophilic and aromatic, creating many face-to-face aromatic interactions with the aromatic rings of remdesivir.
